# Microcapsules Consisting of Whey Proteins-Coated Droplets of Lipids Embedded in Wall Matrices of Spray-Dried Microcapsules Consisting Mainly of Non-Fat Milk Solids

**DOI:** 10.3390/foods10092105

**Published:** 2021-09-06

**Authors:** Minghua Wang, Yael Rosenberg, Moshe Rosenberg

**Affiliations:** Department of Food Science and Technology, University of California Davis, One Shields Ave., Davis, CA 95616, USA; Ming.Wang@conagra.com (M.W.); yrosenberg@ucdavis.edu (Y.R.)

**Keywords:** microencapsulation, oxidative stability, surface excess, lipids, protein adsorption

## Abstract

The effects of wall composition and heat treatment on the formation and properties of core-in-wall emulsions (CIWEs) consisting of whey protein-coated milkfat (AMF) droplets and a dispersion of non-fat milk solids (MSNF) were investigated. Microcapsules were prepared by spray drying these CIWEs. The d_3.2_ of the CIWEs ranged from 0.36 to 0.54 μm. Surface excess of the CIWEs ranged from 1.39 to 6.57 mg/m^2^, and was influenced by concentration of whey proteins and heat treatment (30 min at 90 °C). Results indicated a preferential adsorption of β-lg at the O/W interface. Whey proteins accounted for up to 90% of the proteins adsorbed at the O/W interface. The core retention during spray drying ranged from 90.3% to 97.6% and microencapsulation efficiency ranged from 77.9% to 93.3%. The microcapsules exhibited an excellent long-term oxidative stability at 20 and 30 °C that was superior to that of microcapsules consisting of milkfat and MSNF, where the O/W interface was populated mainly by caseins. The superior oxidative stability could be attributed to the formation of dense whey-proteins-based films at the O/W interfaces of the CIWEs that isolated the core domains from the environment. The results open new opportunities in developing highly stable lipids-containing microcapsules and dairy powders.

## 1. Introduction

Information about the potential health promoting properties of dietary lipids and, consequently, recommendations calling for enhancing the delivery of such lipids through food have highlighted the challenges that are associated with their delivery through food [1,2]. Many of the dietary lipids are highly susceptible to oxidation and their delivery through food products requires developing approaches for improving their oxidative stability [2,3,4]. Microencapsulation offers an effective means for the entrapment, protection, and delivery of sensitive lipids and related compounds through foods [2,4,5,6,7,8,9]. Different technologies have been successfully applied for the microencapsulation of lipids [6,7,8,9,10,11,12,13] utilizing a broad array of GRAS encapsulating agents (wall materials) consisting of proteins, carbohydrates, gums, and their blends [6,12,13,14,15,16,17,18]. 

Success in the microencapsulation of lipids is governed by the combined influence of a multitude of variables, including: the composition and inherent physico-chemical properties of both the microencapsulating agents (wall materials) and the lipids (core), the core-to-wall mass ratio as well as the colloidal properties, PSD, and stability of the core-in-wall emulsion (CIWE). It is also affected by the composition, physico-chemical, and structural properties of the O/W interfaces in the CIWE, as well as by the structural properties and porosity of the wall matrices [6,9,15,16,19]. 

Both animal- and plant-derived proteins have been successfully utilized as wall materials for lipids microencapsulation [12,14,17,20,21,22,23,24,25,26,27,28]. The physico-chemical and functional properties of caseins (CNs) and whey proteins (WP) have allowed their effective utilization in developing lipids-containing microcapsules [20,26,27,29]. Whey proteins are highly-functional wall materials for the preparation of water-soluble and water-insoluble microcapsules and microspheres containing 10–75% (*w*/*w*. db) [30,31,32,33,34,35]. Anhydrous milk fat (AMF)-containing spray-dried (SD) microcapsules with wall matrices consisting of WPI or blends of WPI and lactose exhibited appreciable long-term oxidative stability at both room and elevated temperature [36]. These microcapsules exhibited molecular-sieve type porosity that was influenced by the core payload [37,38]. The oxidative stability of the encapsulated lipids in those microcapsules was attributed to the presence of very dense WP-based films at the O/W interfaces that effectively hindered gas permeation across the O/W interface, thus protecting the encapsulated lipids against oxidation [36,37,38]. 

Lipids-containing spray-dried (SD) dairy powders, such as whole milk powder (WMP) and filled milk powders, are important ingredients in dairy and food applications. WMP is the most utilized milk powder; it has an oxidative shelf life of about 6 months, and a need to develop a new generation of milkfat-containing dairy powders with enhanced oxidative stability exists. In light of the findings of Moreau and Rosenberg [36,37,38], it can be hypothesized that SD microcapsules consisting of WP-coated milkfat droplets embedded in wall matrices consisting of mainly caseins and lactose can be expected to exhibit an oxidative stability that is superior to that of dairy powders that are prepared by SD of homogenized milk or recombined milk. Proving the validity of this hypothesis would allow introducing an advanced milkfat-rich SD powder with enhanced oxidative stability. However, the interfacially-adsorbed protein layer that is adsorbed at the O/W interfaces in homogenized whole milk or recombined whole milk consists mainly of caseins [39,40,41]. Additionally, in the presence of caseins, WP that are already adsorbed at O/W interfaces can be displaced by caseins [40]. 

Introducing and maintaining the WP dominance at the O/W interface of CIWE presents a challenge that, potentially, can be overcome by heat-treating WP-stabilized CIWE in a way that will cross-link the interfacially adsorbed WP by virtue of disulfide bonds. Thus, the objectives of the research were: to investigate and develop CIWE consisting of WP-coated milkfat droplets dispersed in a continuous phase containing mainly caseins and lactose; to investigate the effects of composition and heat treatment on the composition of the interfacially adsorbed protein layer at the O/W interface; and to prepare model microcapsules by SD the investigated CIWE and to study their properties and oxidative stability. 

## 2. Materials and Methods

### 2.1. Materials

Whey protein isolate (WPI) containing 95.6% protein, 1.84% ash, and 2.68% moisture was purchased from Davisco Food Ingredients International (Minneapolis, MN, USA). Anhydrous milkfat (AMF) containing 99.8% milkfat was purchased from Grassland Dairy Products (Greenwood, WI, USA). Low-heat nonfat milk powder containing 0.7% milkfat, 34.49% proteins, 54.93% carbohydrates, 6.3% ash, and 3.5% moisture was purchased from Crystal Cream & Butter Co. (Sacramento, CA, USA), and served as a source for non-fat milk-solids (MSNF). Tris-HCl, SDS, glycerol, bromophenol blue, β-mercaptoethanol, SDS-PAGE molecular weight marker proteins (low range), g acrylamide + g bis-acrylamide, and Coomasie Blue R-250 were purchased from Bio-Rad (Richmond, CA, USA). Acetic acid, methanol, purified (>90%) individual caseins and whey proteins, petroleum ether (analytical grade, bp 70 °C), and n-Hexanal (>98%) were all purchased from Sigma Chemical Company (St. Louis, MO, USA).

### 2.2. Microencapsulation by Spray Drying

WPI solutions containing 1–5% (*w*/*w*) WPI were prepared in de-ionized water (Millipore, 18.2 MΩ.cm) at 50 °C, their pH was adjusted to 6.8, using 0.1 M HCl or 0.1 M NaOH, and AMF (at 50 °C), and they were then emulsified into the WPI solutions at a proportion of 50% (*w*/*w*, db). The emulsification was carried out using the previously reported two-step process [23,42]. In short, a coarse emulsion was prepared using an Ultra-Turrax T25 homogenizer (IKA Works, Cincinnati, OH, USA) operated at 13,000 rpm for 45 s at 50 °C. Then, the coarse emulsion was subjected to four successive homogenization steps (50 MPa) using a model NS1001L2K Panda high-pressure homogenizer (Niro Soavi S.p.A., Parma, Italy). The temperature of the emulsion was maintained at 50 °C throughout the emulsification process. The CIWEs that were prepared in this manner were designated as non-heat-treated (NH) base emulsions (BE) and the WPI concentration in the WPI solution that had been utilized for preparing the BE was included in the designation. For example, NH BEs that were prepared with WPI solutions containing 1.5% or 5% WPI were designated NH1.5 and NH5.0 BEs, respectively. In order to investigate the influence of heat treatment on emulsion properties, beakers containing BEs that had been prepared as described above were placed in a temperature-controlled water bath and were heat-treated at 90 °C for 30 min while being stirred at 150 rpm using a Model 50000-30 Mixer Head (Servodyne Inc., Chicago, IL, USA) connected to a stirring-rate control unit. The heat-treated BEs were designated HT1.0, HT1.5, HT2.0, HT2.5, and HT5.0 BEs, for emulsions prepared with WPI solutions containing 1.0%, 1.5%, 2.0%, 2.5%, and 5.0% WPI, respectively. Final CIWEs (FE) were prepared by combining each of the NH and HT BEs with a dispersion of MSNF in de-ionized water at 50 °C. The final emulsions were designated with the letter “F” according to the BE that has been used in their preparation. For example, final CIWEs that were prepared with NH1.5 or with HT2.5 were designated FNH1.5 and FHT2.5, respectively. In all cases, the lipids content of the FEs was adjusted to 43% (*w*/*w*) and the total concentration of wall solids (WPI plus MSNF) was adjusted to 20% (*w*/*w*). A control CIWE was prepared by emulsifying AMF, to a final core load of 43%, into a 20% (*w*/*w*) aqueous dispersion of MSNF, using the above-detailed two-step homogenization procedure. The CIWEs were spray dried using an APV Anhydro Laboratory Spray Dryer (APV Anhydro A/S Søborg, Denmark). In all cases, atomization was carried out using the centrifugal atomizer of the dryer operated at 50,000 rpm, and drying was carried out (in the co-current configuration) at an inlet and outlet air temperature of 160 ± 2 °C, and 80 ± 2 °C, respectively. The dry microcapsule powders were placed in hermetically closed glass jars and kept in desiccators pending analyses. The dry powders were designated according to the final CIWE that had been used for their preparation. 

### 2.3. Analyses

#### 2.3.1. Particle Size Distribution

The particle size distribution (PSD) properties of the BEs and FEs were investigated using a Malvern Mastersizer MS20 (Malvern Instruments, Malvern, England). The analysis was carried out in quadruplicates using a 2-mW He-Ne laser beam (633 nm) and a 45-mm focus lens. In all cases, the PSD, mean particle diameter (d_3,2_ μm), and the specific surface area (SSA m^2^/mL) of the investigated CIWE were recorded.

#### 2.3.2. Surface Excess

The protein load per unit surface area of the O/W interface and surface excess (Г, mg/m^2^) were determined using the procedure that has been reported earlier [23,42]. Briefly, proteins that were not tightly engaged at the O/W interface of the investigated CIWEs were removed by a series of three successive cream separation and washing steps, leaving at the O/W interface of the investigated “separated washed creams” (SWC) only proteins that were either directly adsorbed at the O/W interface or were tightly bound to proteins that were adsorbed at the interface [23,42,43]. The protein and lipids content of the SWC was determined using the methods that are described below. 

#### 2.3.3. Protein and Fat Content

The total protein content (N × 6.25) of the SWC was determined, in quadruplicates, using the Macro-Kjeldahl test procedure [23,44]. The total AMF content of the SWC and of dry microcapsules was determined in quadruplicates, using a modification of the Roese-Gottlied test procedure, as has been previously reported [23,24]. Using the determined SSA, the results of the AMF and protein content of the SWC, and using an average value of AMF density at 20 °C of 0.935 g/mL, the surface excess was calculated according to Equation (1) [23].

(1) $$\Gamma =\frac{P}{\frac{O}{0.935}\;\times \;\mathrm{SSA}}$$

where: Г is surface excess (mg/m^2^), P and O are protein and oil content in washed cream (mg/g), respectively, and SSA is the specific surface area of CIWE (m^2^/mL). 

#### 2.3.4. Composition of Proteins Adsorbed at the O/W Interface

The composition of the interfacially adsorbed proteins in the SWC was determined by SDS-PAGE, according to the method described by Euston et al. [45]. Wet cream containing about 0.1 g dry solids was dispersed into 2.5 mL of sample buffer consisting of Tris-HCl (0.06 M), SDS (5 *w*/*v*%), glycerol (10 *v*/*v*%), bromophenol blue (0.02 *w*/*v*%), and β-mercaptoethanol (5 *v*/*v*%). The mixture was heat-treated in a boiling water bath for 10 min with frequent vigorous mixing. After cooling to room temperature, 1.5 mL of the dispersion was transferred into a 2-mL test tube, centrifuged at 3000× *g* at room temperature for 15 min, and the aqueous phase was collected for SDS-PAGE analysis. SDS-PAGE was carried out using a Bio-Rad Mini-Protean II Dual Slab Cell (Richmond, CA, USA). The separating gel (0.375 M Tris, pH 8.8) consisted of 15% T ((g acrylamide + g bis-acrylamide)/total volume × 100) and 2.7% C (g bis-acrylamide/ (g acrylamide + g bis-acrylamide) × 100). The stacking gel (0.125 M Tris, pH 6.8) consisted of 4% T and 2.7% C. The electrode buffer consisted of Tris (0.3 *w*/*v*%), glycine (1.5 *w*/*v*%), and SDS (0.1 *w*/*v*%). Ten μL of treated cream sample or of molecular weight marker proteins solution was loaded into each cell and the gels were run at 200 V for 45 min. The gels were stained in a staining solution (0.1 *w*/*v*% Coomasie Blue R-250, 40 *v*/*v*% methanol, and 10 *v*/*v*% acetic acid) for 30 min at room temperature and were then de-stained for 15 min in a solution consisting of 40 *v*/*v*% methanol and 10 *v*/*v*% acetic acid. The remaining background was further de-stained using a solution containing 10 *v*/*v*% methanol and 5 *v*/*v*% acetic acid. The protein bands separated on SDS-PAGE gels were quantified using a laser-enhanced densitometer (Model Ultrascan XL, Pharmacia LKB Biotechnology, Uppsala, Sweden). The scanned data were analyzed, in the 1-D scan evaluation mode, using the Gelscan XL software (Pharmacia LKB Biotechnology, Uppsala, Sweden). Aqueous solution of each of the individual CNs and WPs were treated as described above for the SWC samples and the scanned results were used for constructing a set of linear calibration curves (R^2^ > 0.97) that were used for quantifying the protein composition at the O/W interface of the CIWEs. The results were used to calculate the Protein-Specific Load (PSL, mg/g fat), that is, the amount of a given protein that was adsorbed to the O/W interface that had been created by 1 g of emulsified AMF. The results were also used to calculate the proportion of whey proteins out of total proteins at the O/W interface (WP/TP, %). 

#### 2.3.5. Core Retention

Core retention during spray drying (CR) was defined as the ratio (expressed in %) of core content that was included in 100 g of moisture-free SD microcapsules to that in 100 g of moisture-free CIWE solids [42] and was calculated according to Equation (2).

(2) $$\mathrm{CR}\left(\%\right)=\frac{\mathrm{CEC}}{\mathrm{CEM}}\times 100$$

where: CR is core retention, CEC and CEM are core (AMF) content per unit mass of moisture-free SD microcapsules and CIWE solids, respectively.

#### 2.3.6. Microencapsulation Efficiency (MEE)

The parameter MEE was defined as the proportion (expressed %) of CEC that was not extracted from the microcapsules during 5 min of extraction by petroleum ether and was determined, in triplicates, as previously reported [42]. Briefly, 1 g of SD microcapsules was placed in a 50 mL Quorpak glass bottle (Fisher Scientific, Pittsburgh, PA, USA) into which 25 mL of petroleum ether was added. The extraction was carried out at room temperature for 5 min while gently shaking the dispersions using a Model 360 Garver shaker (Garver Mfg., Union City, IN, USA). Then, the mixture was filtered through a 0.45 µm, 47 mm diameter GN-6 filter (Gelman Science, Ann Arbor, MI, USA); the solvent was evaporated at 70 °C, and the solvent-free extract was dried (45 °C, 6.7 KPa). The dry extracted core (EC) was allowed to reach room temperature in a desiccator and its mass was then determined gravimetrically. MEE was calculated according to Equation (3):

(3) $$\mathrm{MEE}\;\left(\%\right)=\frac{\mathrm{CEC}-\mathrm{EC}}{\mathrm{CEC}}\times 100$$

#### 2.3.7. Oxidative Stability

The long-term oxidative stability of the microcapsules was investigated by monitoring the accumulation of hexanal, one of the most abundant secondary lipid oxidation products [46], during storage at 20 °C and 30 °C for 575 days. The oxidative stability of the microcapsules FHT2.5 and FHT5.0 was also challenged at 50 °C for 90 days. In all cases, the oxidative stability of SD microcapsules that were prepared with the control CIWE was investigated as well at the same storage conditions. Microcapsules samples (about 1.00 g) were placed into 30-mL aluminum foil-wrapped vial (Sunbrokers, Wilmignton, NC, USA). The vials were hermetically sealed with a silicon/PTFE 19 × 3 mm crimp-cap (Phase separation, MI, USA) and were incubated, in the dark, at the different temperatures. The initial level of hexanal in the microcapsules was determined immediately following spray drying, after 24 h of incubation, and then twice a week for samples incubated at 50 °C, and every 20 days for samples incubated at 20 °C or 30 °C. In all cases, independent replicate powders were investigated in triplicates (N × n = 6). 

Hexanal content was determined by solid phase micro-extraction and gas chromatography (SPME/GC). Microcapsule powders were reconstituted with 10 mL of distilled water at 50 °C that was injected into the investigated sample through the vial seal and the mixture was stirred (Vortex-Gene scientific industries Inc., Bohemia, NY, USA Model K-550) for 5 min. Vials were then placed in a water bath at 50 °C, and after equilibration for 5 min, the headspace of the vial was sampled for 20 min at 50 °C using a SPME device with a 100 μm polydimethysiloxane-coated fiber (Supelco Inc., Bellefonte, PA, USA) that was inserted into the headspace. The analytes that had become adsorbed to the sampling fiber were then separated by GC. A Varian (Walnut Creek, CA, USA) model 3400 gas chromatograph equipped with a flame ionization detector (FID), split/splitless injector (Model 1077), and a capillary column (SE-30 30 m × 0.25 mm, 0.25 μm thickness, Alltech Associate Inc., Columbia, MD, USA) was used. The GC conditions consisted of: initial temperature 60 °C, initial hold time 1 min, final temperature 120 °C, final hold time 5 min, and temperature program rate 4 °C/min. The temperature of the injector and detector was maintained at 220 °C and 280 °C, respectively and helium served as carrier gas. Quantification was carried out using a series of linear standard curves (R^2^ > 0.98) that were prepared using mixtures consisting of hexanal (0 to 0.75 ppm, 0.75 to 10 ppm and 10 to 100 ppm), WPI, AMF MSNF, and water. 

#### 2.3.8. Scanning Electron Microscopy (SEM)

The outer topography and inner structure of the investigated microcapsules was investigated using the procedures that have been previously reported [23,24,42]. In short, microcapsules were attached to a double-sided adhesive tape (Ted Pella, Redding, CA, USA) that was placed on a specimen holder. A razor blade was moved perpendicularly through the layer of the mounted microcapsules in order to fracture them and expose their inner structure. The specimens were coated with gold, using a Polaron sputter coater (model E-50050; Bio-Rad, San Jose, CA, USA), and were analyzed using a Philips XL-FEG scanning electron microscope at 5 keV.

#### 2.3.9. Statistical Analysis

The significance of the results was challenged at *p* < 0.05 using the analysis of variance (ANOVA) test procedures that are included in the SigmaStat software (Jandel Scientific Software, San Rafael, CA, USA).

## 3. Results

### 3.1. Particle Size Distribution of CIWEs

**Figure 1:**
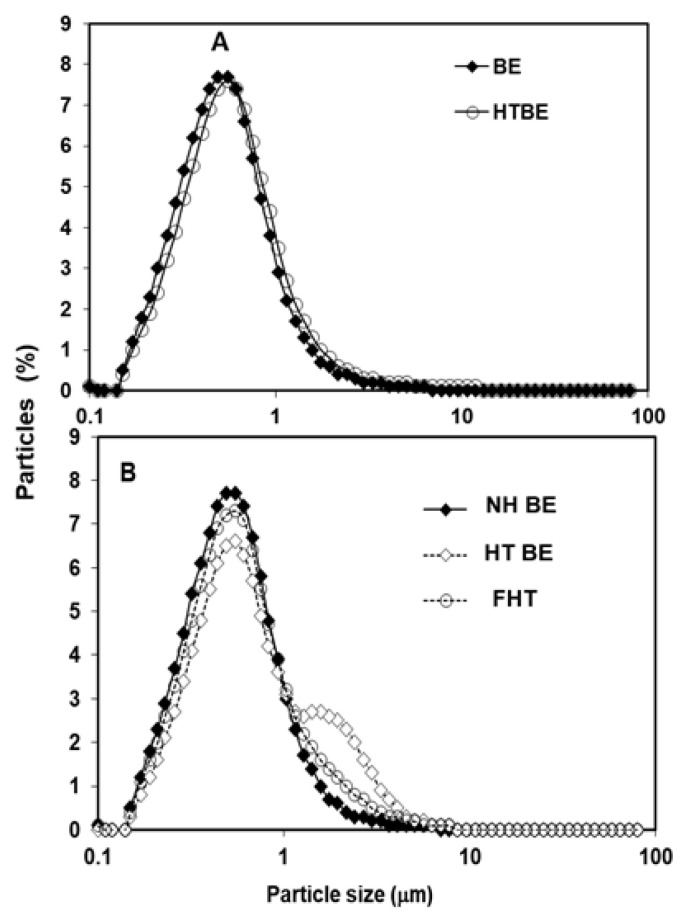
Representative PSD of NH and HT BEs prepared with 1–2.5% WPI (**A**) and PSD of NH5.0, HT5.0, and FHT5.0 (**B**).

**Figure 2:**
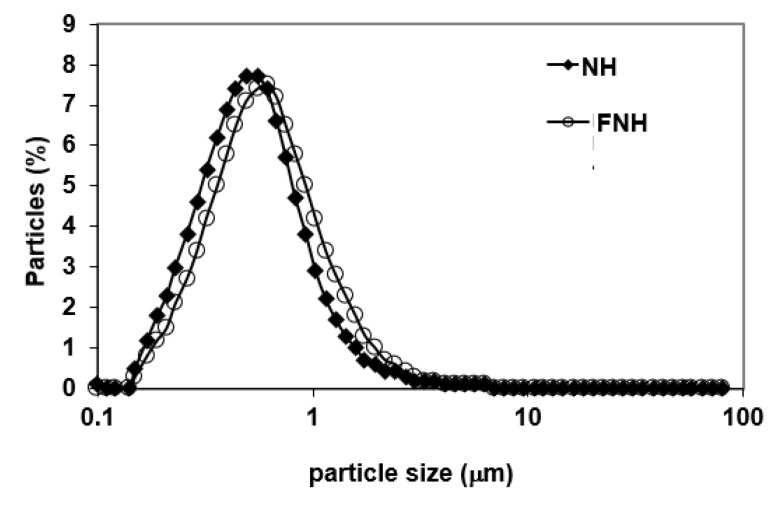
Representative PSD of NH and FNH CIWEs prepared with 1–5% WPI.

Representative PSDs of the investigated CIWEs are depicted in Figure 1 and Figure 2. In all cases, NH BEs and FNH exhibited unimodal PSD. Heat-treated BEs that were prepared with 1–25% WPI and their corresponding FHTs exhibited unimodal PSD. The PSD of HT5.0 BE deviated from unimodality and exhibited a shoulder that could be attributed to the formation of some aggregates consisting of WP-bridged AMF droplets [47]. However, this shoulder was not evident in the FHT50 CIWE (Figure 1), thus indicating that the simple stirring conditions to which HT5.0 BE was subjected during the incorporation of the MSNF dispersed the constituent droplets of these clusters. The latter suggested that these clusters probably consisted of loosely held WP-coated AMF droplets. Results (Table 1) indicated that the d_3,2_ of the investigated BEs ranged from 0.36 to 0.47 μm. The d_3,2_ in FEs that were prepared with BEs containing 1.5–5% WPI ranged from 0.36 to 0.45 μm. Only one FE, FNH1.0, exhibited d_3,2_ that was slightly larger than 0.5 μm (Table 1). In most cases, for a given BE type, d_3,2_ was significantly affected by the WPI concentration (*p* < 0.05). For WPI concentration of 1–2.5%, at a given WPI concentration, NH and of the HT BEs exhibited similar d_3,2_ (*p* > 0.05); however, the d_3,2_ of the HT5.0 BE was larger (*p* < 0.05) than that of the NH5.0 (Table 1). Results thus indicated that, in agreement with what has been previously reported [47], heat treating the BE had a limited influence on the mean particle size of the investigated CIWEs. NH BEs prepared with 1–5% WPI and HT BEs prepared with 1–2.5% WPI exhibited a unimodal particle size distribution (Figure 1). It has to be mentioned that, although not measured in this research, the viscosity of the heat-treated CIWEs was slightly higher than that of the NHs. However, the latter did not adversely affect the atomization of the CIWE during SD. Comparing the d_3,2_ of a given BE to that of its corresponding FE (Table 1) indicated that, in all but two cases, the d_3,2_ of the FE was either similar (*p* > 0.05) or only slightly larger than that of the corresponding BE (*p* < 0.05). The control CIWE exhibited PSD properties similar to those of the investigated CIWEs, having a unimodal PSD with a d_3,2_ of 0.47 μm.

### 3.2. Surface Excess and Protein-Specific-Load

Information about the protein-based interfacially adsorbed layer at the O/W interfaces of the investigated CIWEs is provided by the results of the surface excess (Г) and protein-specific load (PSL) analyses (Table 1 and Table 2). These analyses were carried out using SWCs and thus the Г and PSL results (Table 1 and Table 2) represent only proteins that were strongly engaged at the O/W interfaces of the investigated CIWEs [23,39]. 

For both NH and HT BEs that were prepared with a WPI solution containing 1–2.5% WPI, the within-BE type differences in Г were small, and, in most cases, insignificant (*p* > 0.05); however, at 5% WPI, the differences were significant (*p* < 0.05). The Г of NH5.0 and HT5.0 was 2.1 times higher than that of the NH1.0 and HT1.0, respectively. The surface excess was significantly affected by the heat treatment and in all cases, Г of HT BEs was higher (*p* < 0.05) than that of the NH BEs. The surface excess of the NH and HT BEs ranged from 1.39 to 2.90 mg/m^2^ and from 2.24 to 4.73 mg/m^2^, respectively. The Г of the HT BEs was 1.2–1.6 times higher than that of the corresponding NH BEs. The Г of the NH CIWEs and that of the HT CIWES, except for the HT5.0 CIWE, was lower than that reported for emulsions stabilized by milk protein isolate but higher than that reported for emulsion stabilized by sodium caseinate [43]. The Г of the NH1.0 BE was similar to that reported by Jimenez-Flores et al. [47] for emulsion stabilized by 1% WPI; however, that of HT1.0 BE was higher than what was reported by Jimenez-Flores et al. [47] for heat-treated emulsion stabilized by 1% WPI. 

The surface excess of the BEs reflected the adsorption of WP onto the O/W interfaces during the emulsification/homogenization processes that resulted in the formation of a very dense “base-layer” consisting of whey proteins [48,49,50,51,52,53]. The Г of the HT BEs also reflected the influence of post-homogenization heat-induced protein-protein interactions. Such interactions included SH/SS exchange reactions and non-covalent interactions, such as hydrophobic interactions and hydrogen bonding among the protein constituents of the base layer. Additionally, these interactions also involved WP constituents of the base layer and WP or WP-based structures, such as molecular aggregates, from the bulk phase of the heat-treated emulsion [51,54,55,56]. Overall, the heat-induced protein-protein interactions in the HT BEs cross-linked, and thus significantly stabilized, the interfacially-adsorbed protein-based layer and also resulted in the formation and stabilization of protein-based multi-layer structures at the protein-coated O/W interface [47,51,54,55,56,57,58]. It has to be noted that a certain extent of protein-protein interactions among the unfolded and interfacially-adsorbed WPs at the O/W interfaces of NH BEs could also be anticipated. The latter likely involved unengaged active SH groups of β-lg as well as non-covalent interactions [52,53,56,57]. 

In all cases, Г of the final emulsions (Table 1) was higher than that of the BEs. The Г of the FEs ranged from 2.91 to 3.75 mg/m^2^ and from 2.80 to 6.57 mg/m^2^ for the investigated FNHs and FHTs, respectively (Table 1). Overall, Г of the FEs was 1.16 to 2.70 and 1.21 to 1.81 times higher than that of the BEs from which they had been prepared for FNH and FHT CIWEs, respectively. The Г of the FEs reflected the combined influence of protein-protein interactions at the WP-coated O/W interface of the BE that involved already adsorbed WPs, unengaged WP constituents of the BE as well as WP- and CN-constituents of the added MSNF. Potentially, such interactions included adsorption of WPs and CNs at the WP-covered O/W interfaces as well as, potentially, protein-specific displacement of already-adsorbed WP from the interface by CNs or WPs [51,56,57,58]. 

Results (Table 2) indicated that in all cases, the compositions and process conditions that were used for preparing CIWEs allowed the adsorption and stabilization of very significant proportions of WP at the O/W interfaces that remained stable in the presence of caseins (Table 2). The WP constituents of the interfacially-adsorbed layers accounted for 33.8% to 75% and 45.4% to 90.5%, of the total protein constituents of the interfacially-adsorbed layers in FNH and FHT CIWEs, respectively. Results (Table 2) indicated that when NH and HT BEs were prepared with WPI solutions containing more than 1.5% or 1.0% WPI, respectively, the proportion of WP that became strongly engaged at the O/W interfaces of the FEs was larger than that of the adsorbed caseins. This observation agreed with what was previously reported [51] for emulsions stabilized by WPI of β-lg into which caseins were added. 

The protein-specific loads (PSL) at the O/W interfaces that were associated with 1 g of emulsified AMF are depicted in Table 2. The PLS of both α-lac and β-lg in the NHs was proportionally related to the concentration of WPI in the emulsion and ranged from 1.79 to 6.39 mg/g AMF and from 11.16 to 27.75 mg/g AMF, respectively (Table 2). In all cases, for a given WPI concentration, the PLS of α-lac in the NHs was 41–92% higher than that in corresponding FNHs. For β-lg, the opposite was the case and its PSL in the FNHs was 31% to 92% higher than that in the BE from which it had been prepared (Table 2). The β-lg PSL in the NH BEs was 4.34 to 7.02 times higher than that of α-lac, while in FNHs, the β-lg PSL was 8.12 to 20.23 times higher than that of the α-lac. These results could be attributed to the combined influence of preferential adsorption of β-lg at the O/W interface and to some displacement of α-lac by β-lg at the O/W interface [47,59]. The significant increase in the β-lg PSL that was exhibited by the FNHs also suggested adsorption of WP from the added MSNF onto the already established WP-populated O/W interface of the BEs. The α-lac PSL in the HTs and in the FHTs ranged from 1.93 to 5.79 mg/g AMF and from 1.86 to 10.7 mg/g AMF, respectively (Table 2). The α-lac PSL in the HT5.0 and in the FHT5.0 was significantly higher than that in all other CIWEs of this type (*p* < 0.05). For WPI concentration of 1–2.5%, the among-HTs and the among-FHTs differences in α-lac PSL were very small. The α-lac PSL in FHT5.0, was 1.88 times higher than that in the HT5.0. In all other cases, for a given WPI concentration, the α-lac PSL in the HT CIWE was only slightly higher than that in the FHT. The results suggested that for an initial WPI concentration of 1–2.5%, the heat treatment stabilized the α-lac at the O/W interface of the HT BEs in a way that prevented its displacement from the interface by either β-lg or caseins. The significantly higher α-lac load that was exhibited by the FHT5.0 probably also reflected the engagement, at the O/W interface, of molecular aggregates consisting of the two major whey proteins [60]. 

The β-lg PSL in the HTs and in the FHTs was proportionally related to the initial WPI concentration and ranged from 15.27 to 39.79 mg/g AMF and from 27.68 to 61.49 mg/g AMF, respectively (Table 2). In all cases, the β-lg PSL in the HT and in the FHT CIWEs was significantly (*p* < 0.05) higher than that in the corresponding NH and FNH CIWEs, respectively (Table 2). These results suggest the preferential adsorption of β-lg at the O/W interfaces of the FHT CIWEs and the formation of stable β-lg-rich structures at these interfaces [47]. The preferential adsorption of β-lg at the O/W interface of heat-treated CIWEs can be attributed to its high hydrophobicity at the heat-induced unfolded state as well as to the exposure and activation of its SH group [59]. The high β-lg load in the FHT CIWEs could probably be attributed to interactions, at the O/W interfaces, that involved un-engaged, unfolded, and activated β-lg molecules, and active β-lg-rich molecular aggregates that resulted from the heat treatment stage, as well as β-lg from the MSNF. Collectively, these active constituents were engaged in protein-protein interactions at the O/W interfaces of the emulsions that resulted in the formation and stabilization of β-lg-rich structures at the O/W interfaces of the FHT CIWEs. 

In all cases, the overall CNs PSL in the FEs was inversely proportional to the initial WPI concentration and ranged from 13.54 to 43.53 mg CN/g AMF and from 7.60 to 35.60 mg CN/g AMF for the FNH and FHT CIWEs, respectively. In all cases, the CNs PSL of the FHT CIWEs was significantly lower than that of the FNH CIWEs (*p* < 0.05). The proportion of WP that populated the O/W interfaces of final emulsions ranged from 33.8% to 75% and from 45.4% to 90.5% for the FNH and FHT CIWEs, respectively (Table 2). Overall, the results indicated that the proportion of WP that populated the O/W interfaces in the final emulsions could be successfully modulated by adjusting the initial WPI concentration in the wall solution and by introducing a very significant extent of heat-induced protein-protein interactions that stabilized high proportions of WP at the O/W interfaces. The results indicated that heat treating the base emulsion resulted in the formation of stable WP-based structures at the O/W interface of the BE that were not susceptible to a significant extent of displacement by caseins. Results indicated that Г of the control CIWE was 7.42 mg/m^2^. As expected [40], the interfacially adsorbed protein layer in this CIWE consisted of >96% caseins and only very small proportions of WP.

### 3.3. Microstructure, Core Retention and Microencapsulation Efficiency

**Figure 3:**
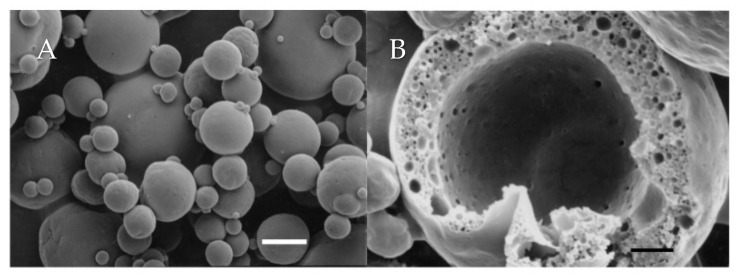
The typical outer topography (**A**) and inner-structure (**B**) of spray-dried microcapsules prepared with FNH 2.5 CIWE. Bar = 10 μm and 4 μm for (**A**,**B**), respectively.

The typical outer topography and inner structure of the investigated microcapsules is depicted in Figure 3. In all cases, the dry microcapsules exhibited only a limited extent of surface indentation and were characterized by a high extent of physical integrity. The outer surfaces of the microcapsules were free of visible cracks or pores and, in general, the microcapsules exhibited the typical structural features of spray-dried microcapsules consisting of milk-derived solids [32,52]. Analyzing the inner structure of the microcapsules revealed that the core (AMF) domains were evenly distributed throughout the wall matrices and were physically isolated from the outer environment (Figure 3B). Results indicated that, in agreement to what has been described before for microcapsule prepared with WPI-stabilized CIWEs [32], the AMF droplets were each coated with dense interfacially-adsorbed protein-based films (Figure 3B). The structural details of the microcapsules did not reveal the presence of pores or cracks connecting the core domains with the outer surfaces of the microcapsules. 

The core retention during spray drying of the investigated CIWEs was high and ranged from 90.3% to 97.3% (Table 3), and the core retention of the control microcapsules was 96.3%. Small among-microcapsule-powders differences in the extent of core retention during spray drying were found (Table 3); however, these differences did not exhibit any specific trend with respect to any of the investigated compositional or physico-chemical properties of the CIWEs. The level of core retention that was attained with the investigated SD CIWEs was similar to that reported for the encapsulation of AMF in wall matrices consisting of WPI or blends of WPI and carbohydrates [61,62]. It has been established that core retention during SD of CIWEs is mainly affected by the PSD of the dispersed phase, the core-to-wall ratio in the CIWE, drying properties of the bulk phase of the CIWE, atomization and drying properties, PSD of the atomized CIWE droplets, as well as the inherent physico-chemical properties of the wall constituents [24,42,61,62,63]. Core losses during SD of CIWEs are significantly affected by the proportion of core droplets that reside at the surface of the atomized CIWE droplets, immediately after atomization, and by the rate and extent to which core droplets arrive at the drying surface of these droplets, due to the internal mixing, prior to the formation of dry crust and a steep viscosity gradient from the surface of the drying droplets to its interior [10,23,42,61,62]. The total solids content of FEs as well as the core-to-wall ratio in these CIWEs were kept consistent. The investigated CIWEs exhibited similar PSD properties (Table 1), and SD was carried out at consistent atomization and drying conditions. The denaturation of WP during the preparation of the HT BEs could have enhanced the water-holding capacity of the WP constituents of BE and, thus, could have had some effect on the drying rate and, consequently, core retention. Additionally, the formation of some complexes between un-engaged β-lg and caseins, especially, k-CN, in the bulk phase of the FHT CIWEs, could have affected the viscoelastic and drying properties of these CIWEs. The among-CIWEs differences in core retention could probably be attributed to the combined effect of these phenomena [23,24,42,43]. 

Microencapsulation efficiency (MEE) of the investigated microcapsules ranged from 77.9% to 93.3% and from 86.6% to 90.8% for microcapsules consisting of SD FHT and FNH CIWEs, respectively, and the MEE of the control microcapsules was 96.1%. In most cases, for a given type of CIWE, MEE was affected by the initial concentration of WPI in the wall solution (*p* < 0.05). Microcapsules prepared with FHT2.5 and FHT5.5 CIWEs exhibited the overall highest MEE (91.3% and 93.3%, respectively) among the investigated CIWEs. At a given set of extraction conditions, the proportion of extractable core consists of core that is truly extracted from the outer surface of the dry microcapsules and of the proportion of truly encapsulated core that is extracted, by virtue of a leaching process, from core domains that are embedded in the wall matrices [10,23,32]. In light of the short extraction time in the present study, the latter could be assumed to consist of AMF from core domains embedded in the sub-surface wall matrices of the capsules [10,23,32,42,64]. The MSNF contained 54.95% lactose and thus the proportion of lactose in the wall constituent of microcapsules that were prepared with wall solutions containing 1.0% to 5.0% WPI ranged from 49.5% to 39%, respectively. During spray drying, carbohydrates, such as lactose, form a glassy amorphous phase [32,38,42,61]. It has been established that the amorphous lactose that is included in wall matrices of spray-dried microcapsules significantly the diffusion of a non-polar extracting solvent, such as petroleum ether, through the wall matrices, hence limiting the extraction of lipids from truly encapsulated core domains [10,23,32,38,42]. The AMF content of the CIWEs was kept consistently and thus the MEE of the microcapsules reflected the combined influence of the physico-chemical, structural, and porosity properties of the wall matrices as well as the composition of the wall matrices and its drying properties [6,8,9,10,23,24,61]. Additionally, the results suggested that MEE was influenced by the heat treatment. The latter could be possibly attributed to the effects of heat treatment on the aggregation of WP in the bulk phase of the emulsion as well to the formation of some complexes between WP and CN. Collectively, these phenomena could be expected to influence the diffusion of the extracting solvent due to the effects of the heat treatment on the microstructure, porosity and even the overall hydrophobicity of the wall matrices. Yet, additionally, it can be suggested that the afore-detailed increase in Г with WPI concentration, especially in the case of the microcapsules that were prepared with HT CIWEs, also limited the extent to which the extracting solvent could extract AMF from core domains. It was interesting to note that although the proportion of lactose that was included in wall matrices of the microcapsules was inversely related to the proportion of WPI in the wall solution, the highest MEE levels were obtained with microcapsules that were prepared with 2.5% or 5% WPI (Table 3). Similarly, the overall highest MEE was exhibited by the control microcapsules where about 54% of the wall matrices consisted of lactose.

### 3.4. Oxidative Stability

**Figure 4:**
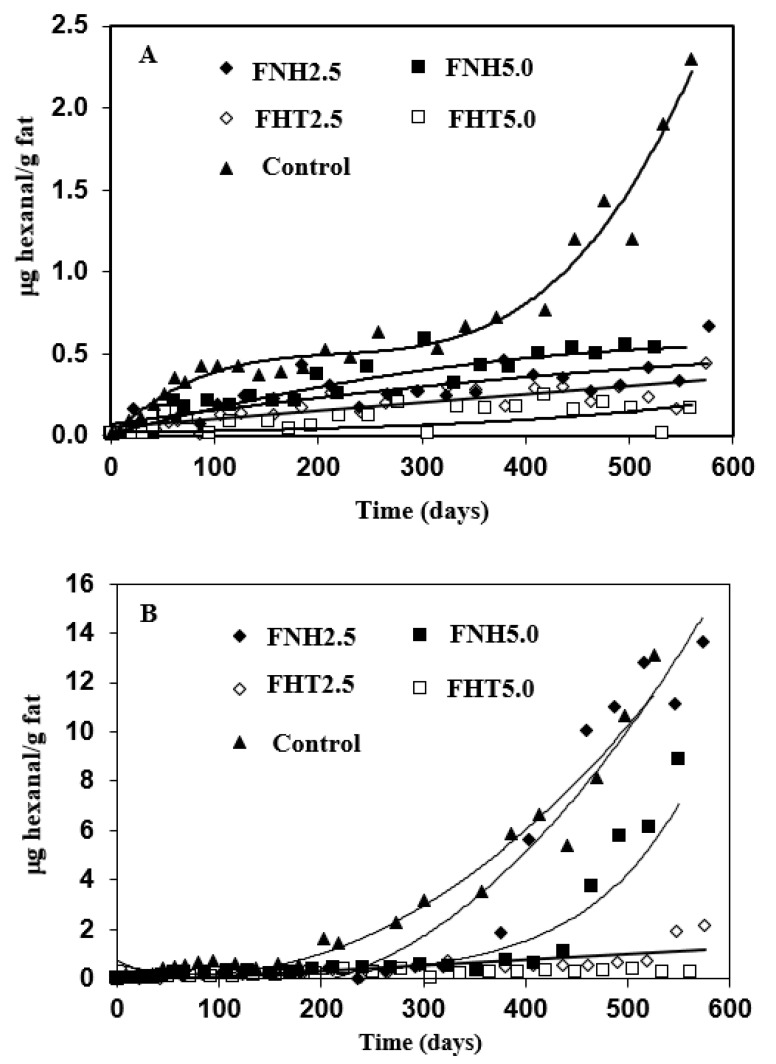
Hexanal accumulation in spray-dried microcapsules consisting of WPI-coated AMF embedded in wall matrices consisting of MSNF during storage at 20 °C (**A**) and 30 °C (**B**). In all cases, AMF content was 43%.

**Figure 5:**
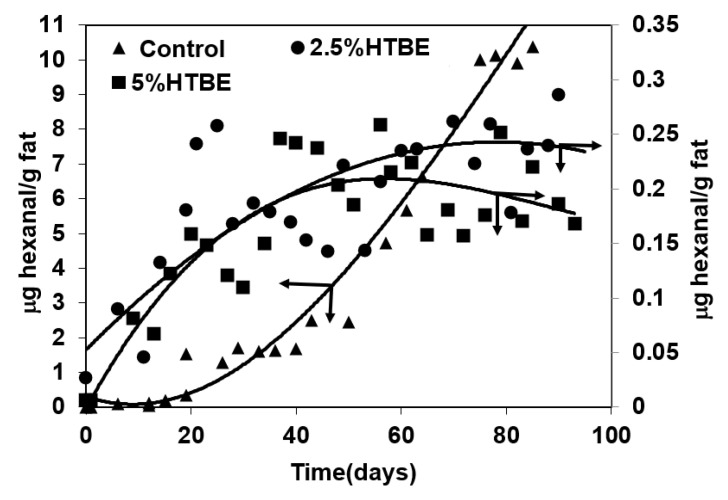
Hexanal accumulation in spray-dried microcapsules consisting of WPI-coated AMF embedded in wall matrices consisting of NFDM during storage at 50 °C. In all cases, AMF content was 43%.

Secondary lipid oxidation products, such as aldehydes and ketones, impact the sensory characteristics of lipids-containing dairy powders [65]. Hexanal is a major product of linoleic acid oxidation; it is involved in the evolution of oxidized flavor in dairy powders and its accumulation has thus been used as an indicator for the oxidative state of the powder [36,66]. Hexanal accumulation during storage is affected by the combined influence of different variables such as storage temperature, fatty acids composition, packaging aspects, presence of metal ions, presence of antioxidants, etc. [65,67]. The odor thresholds of hexanal varies as a function of the medium or matrix in which it is included and it ranges from 0.012 mg/kg in water to 0.32 or 0.075 mg/kg in oil, depending on the sensing mode [66]. The long-term oxidative stability of the microcapsules was therefore investigated by monitoring the accumulation of hexanal during storage at 20 and 30 °C (Figure 4). In all cases, the oxidative stability of the control microcapsule powder, consisting of AMF droplets encapsulated in wall matrices consisting of MSNF, was investigated as well. Results indicated that, except for the control microcapsules, the investigated microcapsules exhibited significant oxidative stability during 575 storage days at 20 °C. After 200 storage days at 20 °C, the level of hexanal in the control powder was 0.5 μg/g fat and continued to increase with storage time, reaching a level of 2.66 μg/g fat after 160 days. Throughout the storage time at 20 °C, the proportion of hexanal that accumulated in all of the other investigated microcapsules was significantly lower than that in the control powder. After 500 days, the level of hexanal that accumulated in the FNH5.0 microcapsules was about 0.5 μg/g fat, while that in microcapsules that were prepared with the FNH2.5 CIWE was lower than 0.3 mg/g fat (Figure 4A). The overall best oxidative stability at 20 °C was exhibited by microcapsules that were prepared with the FHT2.5 and FHT5.0 CIWEs. In both these cases, the level of hexanal that accumulated in the microcapsules remained below 0.5 μg/g fat throughout 550 storage days (Figure 4A). Similar to what was observed at 20 °C, the oxidative stability of the microcapsules stored at 30 °C was affected by the combined influence of the protein composition of the O/W interface and by the heat treatment (Figure 3B). Overall, as could have been anticipated [67], increasing the storage temperature by 10 °C enhanced, to a certain extent, the lipids oxidation. Overall, the poorest oxidative stability at 30 °C was exhibited by the control powder in which 0.55 and 13.1 μg hexanal/g fat accumulated after 70 and 525 days, respectively. The microcapsules that were prepared with the FNH2.5 and FNH5.0 CIWEs accumulated 0.5 μg hexanal after 292 and 280 days, respectively, and after 550 days the level of hexanal in these microcapsules was 13.6 and 8.8 μg/g fat, respectively. Similar to the results of storage at 20 °C, the overall highest long-term oxidative stability at 30 °C was exhibited by the FHT2.5 and FHT5.0 microcapsules (Figure 4B). The level of hexanal in the FHT2.5 microcapsules was 0.5 and 2.17 μg/g fat after 380 and 560 storage days, respectively while its level in the FHT5.0 microcapsules was only about 0.3 μg/g fat after 580 storage days at 30 °C (Figure 4B). The long-term oxidative stability of the microcapsules that were prepared with heat-treated CIWEs was superior to that reported earlier for non-heat-treated microcapsules consisting of WP-coated AMF droplets embedded in wall matrices consisting of WPI or a blend of WPI and lactose [36]. The oxidative stability of the FHT2.5 and FHT5.0 microcapsules was also challenged in an accelerated storage study at 50 °C (Figure 5). Results indicated that after 25 storage days, the level of hexanal in the control microcapsules was about 0.5 mg/g fat and continued to increase with storage time, reaching 10.30 mg/g fat after 80 days. In contrast, the hexanal levels in the FHT2.5 and FHT5.0 microcapsules remained lower than 0.5 mg/g fat throughout the storage at 50 °C (Figure 5). 

The results of the long-term oxidative stability study clearly suggested that the oxidative state of the microencapsulated AMF was noticeably influenced by the surface excess of the CIWEs and by the protein composition of the interfacially-adsorbed protein-based layer (Table 2, Figure 4 and Figure 5). The oxidative stability of microcapsules with AMF droplets that were coated mainly with WP was significantly higher than that of microcapsules with AMF droplets that were coated mainly with CNs. Yet, additionally, the long-term oxidative stability of the encapsulated AMF was proportionally-related to the surface excess and to the WP-PSL (Table 3 and Figure 4 and Figure 5). The oxidative stability was also enhanced by the proportion of PSL of β-lg at the O/W interface, in agreement with a similar effect of this protein that has been reported earlier [59]. In all cases, the overall highest long-term oxidative stability was exhibited by microcapsules that had been prepared with the FHT CIWEs. The latter suggested that the oxidative stability of the encapsulated AMF was markedly enhanced by the formation of tightly-packed and cross-linked, WP-based structures at the O/W interface of the heat-treated CIWEs. The significant oxidative stability of the encapsulated lipids could thus be attributed to the formation of interfacially-adsorbed dense [52] films, consisting mainly of WP, that limited the permeation of oxygen into the core domains, in agreement with what had been suggested by Moreau and Rosenberg [36,37,38]. The superior oxidative stability of the FHT2.5 and FHT5.0 microcapsules suggested that the cross-linked structures at the O/W interfaces of these capsules enhanced the oxidative stability of the encapsulated AMF. The latter can be potentially attributed to a very significant hindrance to oxygen permeation through these interfaces, to an extent that was even higher than that in the microcapsules that were prepared with the FNT CIWEs. The overall highest oxidative stability that was exhibited by the FHT2.5 and FHT5.0 microcapsules probably also reflected the anti-oxidative activity of unengaged sulfhydryl groups of β-lg, both at the O/W interfaces and the bulk phase of these microcapsules [59,67]. 

The control microcapsules exhibited the overall highest MEE, and yet their oxidative stability was the lowest among the investigated microcapsules. The MEE of the FHT2.5 and FHT5.0 microcapsules was higher than that of the corresponding FNH microcapsules (Table 3); however, the small differences in MEE between these microcapsules could not explain the very significant differences in the oxidative stability between microcapsules that were prepared with these CIWEs (Figure 4 and Figure 5). The results of the study thus highlighted the complex nature of parameters that affect the oxidation of microencapsulated lipids. The results of the study indicated that the proportion of surface fat is only one of many other variables that collectively govern the rate and extent of lipids oxidations [68,69]. 

## 4. Conclusions

The results of the study indicated the validity of the research hypothesis. The procedure that was used for preparing the CIWEs allowed establishing and maintaining interfacially adsorbed stable WP-based protein films, consisting of up to 90% WP, even in the presence of caseins in the FEs. The formation and protein composition of the protein films at the O/W interfaces of the CIWEs was governed by the combined influence of the composition of the CIWE and the heat treatment to which the BEs had been subjected. The investigated microcapsules exhibited a long-term oxidative stability that could be attributed to the formation, composition, and cross-linking of the WP-based films at the O/W interfaces of the CIWEs. In addition to the importance of the results to the field of lipids microencapsulation in food applications, the results of the study present new opportunities for preparing lipids-containing dairy powders with oxidative stability that is superior to that of regular whole milk powder. The latter has a special relevance when the effects of the recent COVID-19-related disrupted milk supply chain on the dairy industry are considered. It can be suggested that capabilities to manufacture dairy powders with a long-term oxidative stability represent a tangible alternative to dumping of huge quantities of surplus milk. However, it has to also be recognized that pursuing this opportunity calls for introducing proper legislation that allows modulating the protein composition of milk-derived powders.

## Figures and Tables

**Table 1:** Mean particle size (d_32_, μm) and surface excess (Г, mg/m^2^) in the base (BE) and in the final (FE) CIWE.

BE Type	d_3,2_ (μm)		Surface Excess (mg/m^2^)	
	BE	FE	BE	FE
NH1.0	0.43 ^ALa^	0.54 ^AKa^	1.39 ^CDLb^	3.75 ^AKb^
NH1.5	0.40 ^BLa^	0.43 ^BKa^	1.64 ^BCLb^	3.64 ^BCKa^
NH2.0	0.39 ^BKa^	0.39 ^CKb^	1.77 ^BCLb^	3.26 ^BCKa^
NH2.5	0.37 ^CLa^	0.40 ^CKa^	1.98 ^BLb^	2.91 ^CKb^
NH5.0	0.36 ^CKb^	0.36 ^DKb^	2.90 ^ALc^	3.35 ^BCKc^
HT1.0	0.44 ^BLa^	0.50 ^AKb^	2.24 ^BLa^	4.05 ^BKa^
HT1.5	0.39 ^CDLa^	0.42 ^BKa^	2.45 ^BLa^	3.05 ^CDKb^
HT2.0	0.40 ^CKa^	0.41 ^BKa^	2.32 ^BLa^	2.80 ^DKb^
HT2.5	0.38 ^DLa^	0.41 ^BKa^	2.40 ^BLa^	4.28 ^BKa^
HT5.0	0.47 ^Aka^	0.45 ^BLa^	4.73 ^ALa^	6.57 ^AKb^

^ABCD^ For a given BE type, means in a given column that are followed by different letters differ significantly (*p* < 0.05). ^KL^ For a given tested variable, means in the same raw that are followed by different letters differ significantly (*p* < 0.05). ^abc^ For a given WPI concentration, means in a given column followed by different letters differ significantly (*p* < 0.05).

**Table 2:** Protein composition at the O/W interface of base (BE) and final CIWEs. Content of α-Lac (mg/g fat) and β-Lg (mg/g fat) and caseins (CNs, mg/g fat).

BE Type	α-Lac (mg/g Fat)		β-Lg (mg/g Fat)		CNs (mg/g Fat)	WP/TP (%)
	BE	FE	BE	FE		
NH1.0	1.79 ^Ca^	1.06 ^BCb^	11.16 ^Cb^	21.11 ^Bb^	43.53 ^Aa^	33.8
NH1.5	2.40 ^Ba^	1.70 ^CDa^	14.35 ^Cb^	24.65 ^Ba^	28.18 ^Ba^	48.3
NH2.0	2.95 ^Ba^	1.56 ^Cb^	20.71 ^Bb^	25.17 ^Bb^	17.71 ^Ca^	60.1
NH2.5	3.44 ^Ba^	1.79 ^Ba^	21.66 ^Bb^	36.22 ^Ab^	14.85 ^Da^	71.9
NH5.0	6.39 ^Aa^	4.46 ^Ab^	27.75 ^Ac^	36.23 ^Ab^	13.54 ^Ea^	75.0
HT1.0	1.93 ^Ba^	1.89 ^Ca^	15.27 ^Da^	27.68 ^Da^	35.60 ^Ab^	45.4
HT1.5	2.11 ^Bb^	1.86 ^Ca^	19.33 ^Ca^	27.62 ^Da^	14.95 ^Bb^	66.3
HT2.0	1.98 ^Bb^	1.98 ^Ba^	24.50 ^BCa^	38.77 ^Ca^	14.66 ^Bb^	73.5
HT2.5	2.06 ^Bb^	1.88 ^Ca^	28.05 ^Ba^	46.98 ^Ba^	12.54 ^Cb^	79.6
HT5.0	5.79 ^Ab^	10.70 ^Aa^	39.79 ^Aa^	61.49 ^Aa^	7.60 ^Db^	90.5

^ABCDE^ For a given BE type, means in the same column that are followed by different letters differ significantly (*p* < 0.05). ^abc^ For a given WPI concentration in BE, means in the same column that are followed by different letters differ significantly (*p* < 0.05).

**Table 3:** Core retention (CR %) during spray drying and microencapsulation efficiency (MEE %) of the spray-dried microcapsules.

FE Type	WPI (%)	CR (%)	MEE (%)
NH1.0	1.0	94.9 ^Bb^	88.1 ^Ba^
NH1.5	1.5	93.8 ^Ba^	87.4 ^Ca^
NH2.0	2.0	97.6 ^Aa^	86.6 ^Da^
NH2.5	2.5	94.4 ^Ba^	88.4 ^Bb^
NH5.0	5.0	97.3 ^Aa^	90.8 ^Ab^
FHT1.0	1.0	96.7 ^Aa^	77.9 ^Db^
FHT1.5	1.5	93.6 ^BCa^	78.9 ^Db^
FHT2.0	2.0	93.0 ^CDb^	86.9 ^Ca^
FHT2.5	2.5	90.3 ^Db^	91.3 ^Ba^
FHT5.0	5.0	96.0 ^Aa^	93.3 ^Aa^

^ABCD^ For a given BE type, means in a given column that are followed by different letter, differ significantly (*p* < 0.05). ^ab^ For a given WPI concentrations, means in a given column that are followed by different letters, differ significantly (*p* < 0.05).

## Data Availability

Not applicable.
